# Sports injuries as reversible involution: a novel approach to rehabilitation and readaptation

**DOI:** 10.3389/fspor.2025.1519404

**Published:** 2025-06-09

**Authors:** Daniel Rojas-Valverde, Emanuel Herrera-González, Diego A. Bonilla

**Affiliations:** ^1^ Centro de Investigación y Diagnóstico en Salud y Deporte (CIDISAD), Escuela Ciencias del Movimiento Humano y Calidad de Vida (CIEMHCAVI), Universidad Nacional, Heredia, Costa Rica; ^2^ Clínica de Lesiones Deportivas (Rehab & Readapt), Escuela Ciencias del Movimiento Humano y Calidad de Vida (CIEMHCAVI), Universidad Nacional, Heredia, Costa Rica; ^3^ PSICOMI, Escuela Ciencias del Movimiento Humano y Calidad de Vida (CIEMHCAVI), Universidad Nacional, Heredia, Costa Rica; ^4^ DBSS Research Division, Dynamical Business Science Society–DBSS International SAS, Bogotá, Colombia; ^5^ Grupo de Investigación NUTRAL, Facultad Ciencias de la Nutrición y los Alimentos, Universidad CES, Medellín, Colombia; ^6^ Hologenomiks Research Group, Department of Genetics, Physical Anthropology and Animal Physiology, University of the Basque Country (UPV/EHU), Leioa, Spain

**Keywords:** recovery of function, sports medicine, exercise therapy, physiotherapy, sports science, athletic training: return to play, reconditioning, allostasis

## Abstract

This perspective article discusses the notions of reversible involution in sports injuries and offers an innovative operational framework for rehabilitation that we expect to help with the athlete's readaptation process. Injuries have traditionally been managed by relieving the symptoms and recovery, but physiological regulation based on the allostasis-interoception model suggests that these injuries are dynamic and reversible. This reconceptualization leads to a holistic approach beyond recovery, allowing athletes to have an optimally functioning physiology. The model emphasizes on individualized rehabilitation and readaptation approaches considering the complexity of sports injuries. Under the proposed framework, rehabilitation involves treating the immediate consequences of injury and attending to physical, physiological, psychological, technical, and tactical changes by encouraging holistic attention. By treating sports injuries as reversible involution, this framework supports a multifaceted approach to rehabilitation and readaptation, where recovery relates to physiological changes reflecting interactivity between an individual's potential for healing. This approach aims to move beyond the fixed paradigms in sports medicine by providing a multidimension vision for optimizing the process of rehabilitation to accelerate athletes’ health and well-being after injuries during sport-related activities.

## 1 Introduction

Athletes often face sports-related injuries, which can hinder their physical abilities and reduce their competitive edge. In this regard, recent studies have shed new light on this issue, suggesting that such injuries may be part of a reversible involution process – which involves the regression of structural and functional tissues to an earlier stage, potentially allowing for recovery and restoration (1, 2). Temporary regression of biological systems is a stress-related response that affects individuals physiologically, emotionally, socially, and behaviorally. Within the allostasis-interoceptive paradigm, this phenomenon is commonly referred as cacostasis [“bad state”, from ancient Greek *κακός* (*kakós*) that means “bad”] to refer this state of disharmony and cacostatic load as the cumulative pathophysiological burden of the organism (3).

Sports injuries pose a significant and widespread challenge for athletes across various disciplines. These injuries disrupt training schedules, hinder competitive goals, and lead to profound physical, physiological, and psychological consequences (4, 5). Traditionally, sports injury management has focused on minimizing structural damage and alleviating pain as an effort to speed up recovery. Nevertheless, studying the nature of injuries as a biological outcome within the athletes’ allostatic response (6) presents a transformative perspective that could revolutionize approaches to rehabilitation and readaptation (7–9).

This perspective article explores the intersection between the cacostatic state and tissue involution into the context of sports injuries, proposing a novel operational framework for rehabilitation and readaptation. Through these connections, we aim to promote a practical rehabilitation and readaptation framework for sports injuries. By integrating the concept of reversible involution, we can usher in a new era of sports medicine—one that not only restores athletes to their pre-injury state but also propels them toward higher performance levels. Finally, we also explore the theoretical foundations of this concept and discuss its implications for musculoskeletal injuries, as well as its potential to transform holistic rehabilitation approaches.

## 2 Cacostasis and tissue involution

Within the allostasis-interoception paradigm, allostasis refers to a biological system's ability to adapt to acute or chronic challenges through predictive adjustments to maintain viability (10, 11). Allostasis can be seen as a mechanism from which the body adjusts its physiological parameters within a range that is relatively harmless (e.g., increasing oxygen delivery to muscles during intense exercise, lowering heart rate during post-workout recovery, and redirecting blood flow from the digestive system to active muscles during physical activity), in response to changes anticipated from past experience, aimed at minimizing the future surprise (i.e., free energy). In this context of mathematical analysis and dynamical systems that follows Bayesian inference, allostasis is currently referred to as “variational and relational stability” by Bettinger & Friston (12), which expand upon the traditional concept of “stability through change” and highlight the inherent capacity of organic systems for physiological resilience, or the ability to “return to stability”.

**Figure 1:**
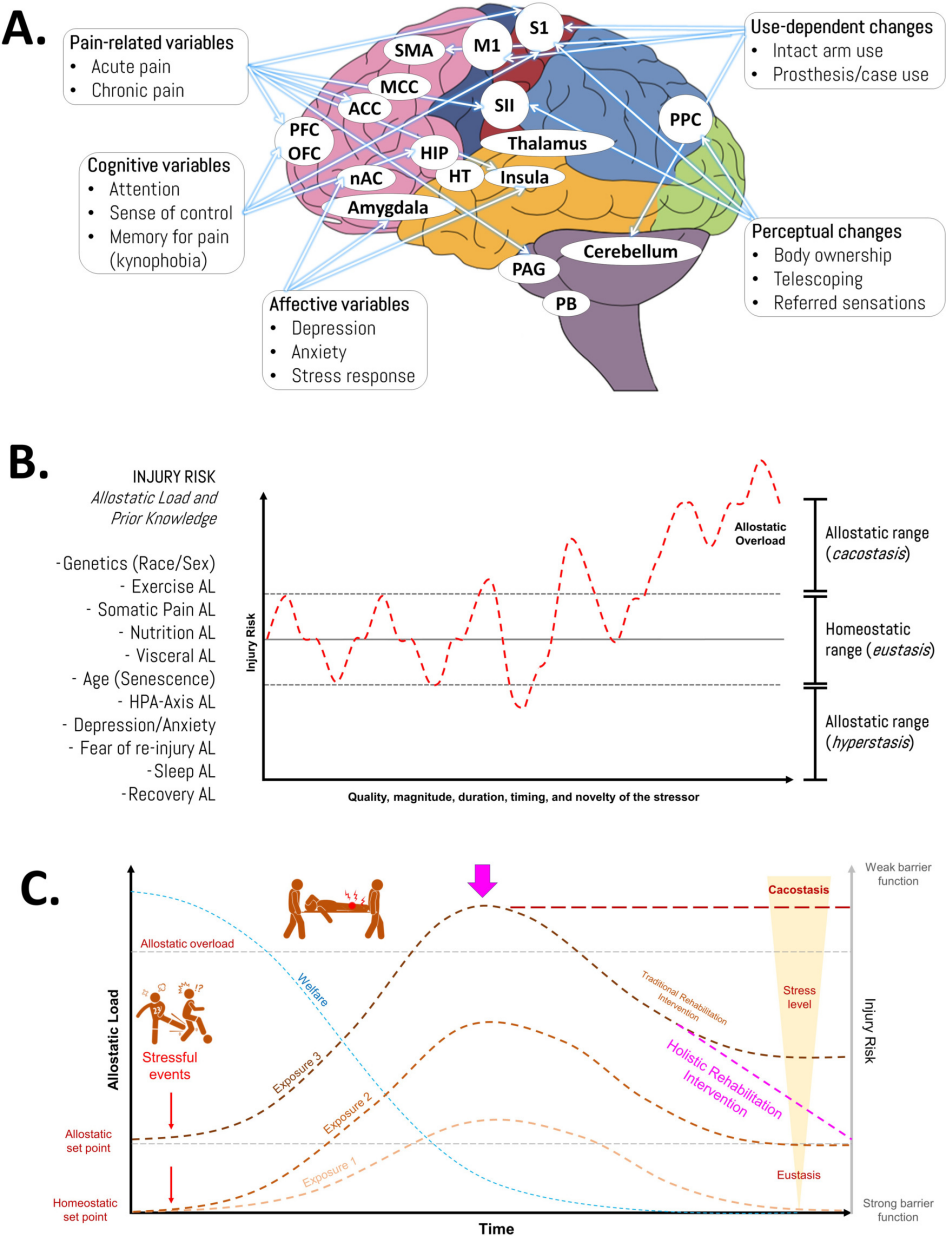
Injuries from an allostatic approach. **(A)** Characteristics of brain reorganization after injuries and musculoskeletal disorders. ACC, anterior cingulate cortex; BG, basal ganglia; HIP, hippocampus; HT, hypothalamus; M1, primary motor cortex; MCC, midcingulate cortex; NAC, nucleus accumbens; OFC, orbitofrontal cortex; PAG, periaqueductal grey; PB, parabrachial nucleus; PCC, posterior cingulate cortex; PFC, prefrontal cortex; PPC, posterior parietal cortex; S1, primary somatosensory cortex; SII, secondary somatosensory cortex; SMA, supplementary motor area. **(B)** Representation of the changes in injury risk in response to stress exposure. Injuries are multifactorial in nature (based on diverse allostatic load types—including genetics, exercise, nutrition, and sleep—along with the athlete's prior knowledge and experiences). **(C)** Schematic illustration of the allostatic overload and its relationship with sports injuries. The figure illustrates how chronic and acute stress contribute to sports injuries through two key physiological benchmarks: the allostatic set point (adapted stress response threshold) and homeostatic set point (baseline equilibrium). During prolonged stress exposure, athletes experience progressive physiological resetting due to accumulated strain, eventually reaching critical cacostasis when injured (pathological imbalance). This state demands immediate holistic rehabilitation interventions post-injury to restore physiological equilibrium and enhance resilience. The framework integrates these concepts to explain stress adaptation/maladaptation in athletic contexts, highlighting the transition from allostatic overload to injury risk and recovery strategies. AL, allostatic load; HPA, hypothalamic-pituitary-adrenal axis. Source: the authors (D.A.B.).

Using sports injuries as an example, acute exercise-related impacts like falls, collisions, or strong tackles (external stressors) can also trigger systemic changes. These exposures recalibrate regulatory parameters to prioritize resource allocation toward activities critical for immediate survival (Figure 1A). The allostatic load, which represents the biological cost of adaptation, can then increase significantly when additional chronic stressors—such as those related to the magnitude, duration, quality, timing, and novelty of the stressor—exceed the system's coping capacity. This leads to a state known as allostatic overload, ultimately resulting in a cacostatic state. In 2022, we proposed this approach to analyze the etiology of pain and common injuries in weight-based resistance training (6) (Figure 1B). Interestingly, recent data validate this as allostatic load correlates with overuse musculoskeletal injuries during 10-week training programs in the US Marine Corps (13). Similarly, Feigel et al. demonstrated that increased allostatic load significantly linked to both physical and psychological maladaptation in military personnel (*n* = 31, 14F) completing a 10-week tactical training course (14).

In general, these processes can lead to tissue involution, characterized by reverse changes in the morphological and physiological features of biological tissues. Inflammation plays a key role in driving tissue involution due to its critical role in cellular responses to injury or stress. For instance, pathways to resolve inflammation are activated, genes related to tissue repair and cytoskeleton remodeling are upregulated, and energy expenditure increases—a phenomenon referred to as “allostasis and stress-induced energy expenditure” (6, 15). Over time, this can impair repair capacity, reducing the ability to regenerate tissues and potentially leading to diminished physical performance (16, 17), particularly due to low energy availability.

In the context of sports injuries, reversible involution refers to a temporary regression in affected tissues—such as muscles, joints, tendons, and ligaments—that disrupt normal function. The underlying basis of injury-induced involution lies in the principle of reversibility within the stress response. When exposed to stressors, the biological system regresses to earlier stages. Therefore, although tissues undergo regressive changes, they can achieve eustasis and return to their previous functional levels. The body's innate repair and regenerative mechanisms support this restorative process. Since the response to stress is dynamic and not permanent, tissue involution can be reversed through well-designed interventions focused on several aspects of healing and recovery (9). Recognizing that injury-induced involution is a transient, rather than permanent, condition allows practitioners to align their methods with the body's natural healing processes.

Rabey & Moloney recommended allostasis as a possible explanatory model for pain onset and persistence, suggesting that incorporating allostatic load into clinical reasoning could enhance decision-making (18). Indeed, in agreement with McClean et al., this underscores the necessity of treating sports injuries through a holistic framework (2). Such interventions may not only restore physiological parameters to eustasis but could also progressively optimize athletic performance reaching the state conceptualized as hyperstasis (3). This shift in perspective encourages the development of targeted systemic strategies (19) to promote tissue regeneration, reduce inflammation, and support reconstruction while preventing cortical changes (e.g., kynesiophobia), optimizing motor control, improving skill training and mental well-being (5) (Figure 1C).

This integration into sports clinical practice lays the foundation for a transformative rehabilitation paradigm, one that addresses not only the physical aspects of injury but also the psychological and emotional dimensions of recovery. By emphasizing reversibility and restoration, we can establish a unified, multidimensional strategy for sports injury rehabilitation and readaptation (7–9). Interestingly, while this exploration into rethinking sports-related injuries is still in its early stages, initial insights into the potential relationship between allostatic overload, the cacostasis state (i.e., tissue involution), and sports injuries (6, 13, 18) suggest a significant shift in the athlete recovery and development paradigm. Reconceptualizing injuries as transient evolutionary states rather than permanent setbacks encourages athletes and coaches to view challenges as opportunities to achieve peak performance.

## 3 Motor development and injury-induced involution

Injury triggers a transitional involution in athletes’ motor development. This state of cacostasis is characterized by regressive factors such as loss of mobility, reduced functional capacity, and suboptimal metabolic responses. Understanding this connection is crucial for recognizing the potential for athletes to recover and adapt as they overcome these temporary setbacks. When athletes sustain injuries, particularly musculoskeletal lesions, they undergo a transitional involution process specific to their motor development. This process involves a temporary decline in motor coordination and physical abilities (20, 21). Transitional involution in motor development reflects a reduction in physical capabilities due to injury-related stressors, alongside physiological changes aimed at restoring lost skills and abilities.

This loss of mobility and functional capacity is one of the distinctive features of injury-induced involution. Athletes cannot move freely and perform even the most basic activities they used to do without difficulty. The physical loss this implies parallels the decay dimensions of cacostasis, during which people briefly lose their ability to handle complicated emotions or manage social situations adequately (13). As mentioned previously, the injury-induced involution affects the metabolic processes of an athlete which might disrupt peak efficiency and substrate utilization. This disturbance can lead to less efficient energy utilization and reduce the athlete's physical performance (14). Hence, nutritional strategies are essential for optimizing rehabilitation and readaptation. As we previously reported (22), key approaches include ensuring adequate energy availability and incorporating diets rich in protein and carbohydrates. Supportive supervision is crucial to prevent low energy availability. Sports nutritionists should aim for a carbohydrate intake of 6–8 g/kg/day to enhance glycogen restoration and reduce metabolic stress, supporting recovery and performance. For persistent muscle soreness or damage, frequent protein intake (4–5 times daily, 0.4 g/kg/meal) can aid repair. Additionally, consuming 25–30 g of protein before sleep may improve overnight recovery and muscle growth. Due to limited controlled clinical trials, definitive conclusions cannot yet be drawn regarding supplements like collagen, vitamin D, HMB, glucosamine, and other micronutrients. However, practitioners should weigh both the potential efficacy and strong safety evidence for creatine monohydrate (23) and omega-3 fatty acids (24) to inform clinical decision-making.

The systematic recovery of motor skills, mobility, and metabolic efficiency enables athletes to not only regain but also surpass their previous fitness levels. Recent neurophysiological advancements have introduced innovative rehabilitation strategies, such as action observation therapy, where patients observe a therapist performing actions to aid recovery (25). In contrast, motor imagery involves mentally simulating muscle actions without physical movement (26). Additionally, the cross-education effect—where training one limb benefits the untrained contralateral limb—has shown promise in injury rehabilitation. This approach, which improves outcomes without directly targeting the injured area, has gained attention for managing unilateral injuries like stroke-induced hemiparesis, osteoarticular injuries, and anterior cruciate ligament injuries (27). These techniques enhance recovery and muscle strength during rehabilitation and can be combined with psychological strategies to boost athletes’ motivation, helping them view injuries as opportunities to become more resilient, adaptable, and successful competitors (5).

## 4 A framework proposal for injury rehabilitation and readaptation

**Figure 2:**
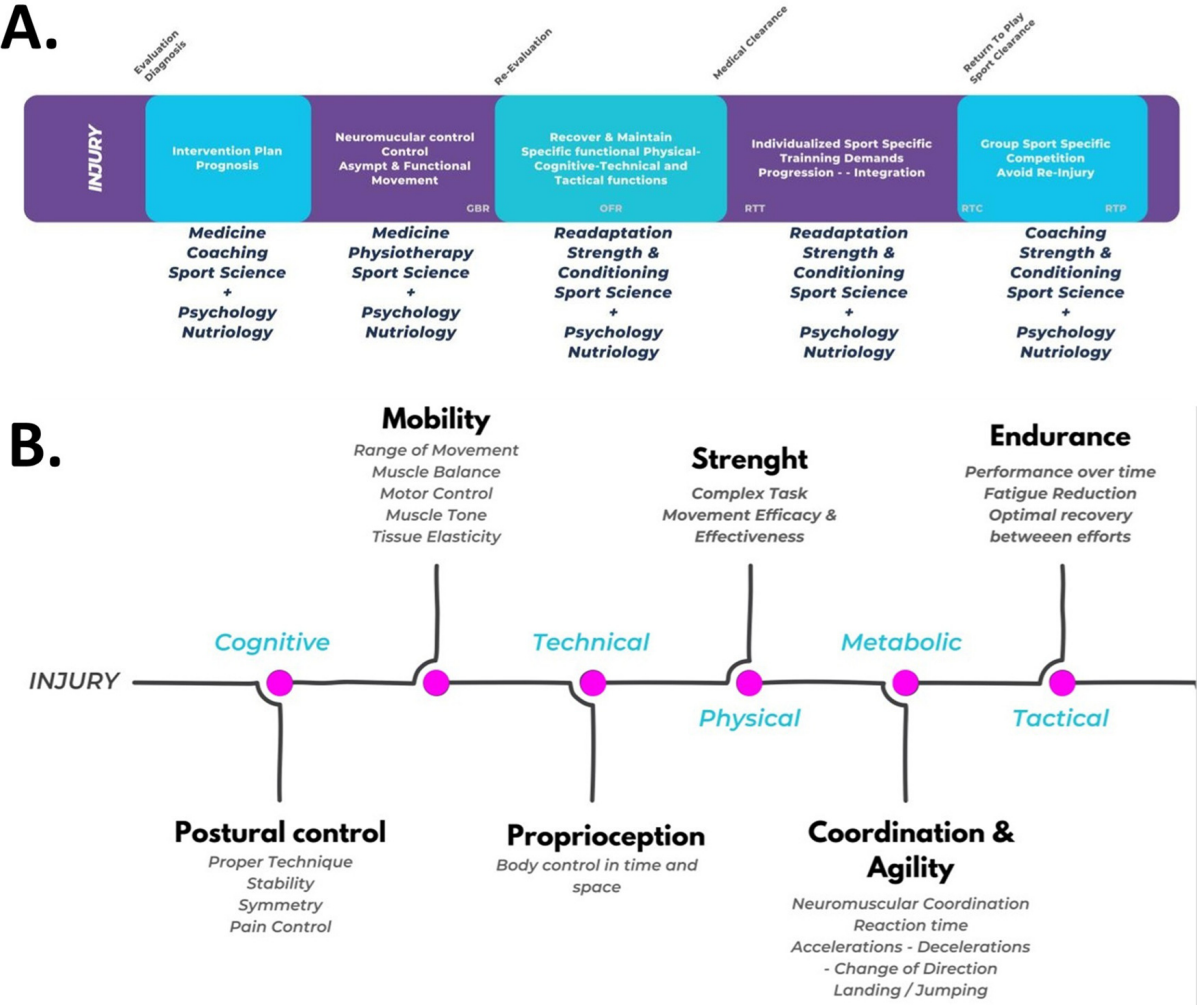
Practical recommendations of the proposed operational framework. **(A)** The flowchart of the sports rehabilitation and readaptation model was adapted from Rojas-Valverde et al. (6). Rehabilitation phases based on (4, 5): GBR, gym-based rehabilitation; OFR, on-field rehabilitation; RTT, return to training; RTC, return to competition; RTP, return to performance. **(B)** Planification model proposal for injury approach: abilities to readapt after reversible involution. Source: the authors (D.R.V.).

The idea of sports injuries as reversible involution introduces a fascinating and transformative paradigm. This regressive tendency, relevant to sports injuries, highlights key factors that can enhance biological recovery toward eustasis and, ultimately, hyperstasis (3). Therefore, a holistic approach is essential for addressing musculoskeletal sports injuries. By examining injuries through the lens of allodynamic responses, practitioners can design personalized rehabilitation programs. Tailoring strategies to the athlete's unique experiences and needs can optimize recovery and support performance improvement. Our proposal is grounded in a well-established framework for rehabilitation and readaptation, widely accepted by coaches, medical staff, trainers, and other sports stakeholders (7–9) (Figure 2A).

Building on theoretical foundations, this framework outlines a comprehensive approach with six key steps to optimize human movement and physical performance. It serves as a valuable guide for designing tailored interventions and training protocols, grounded in theory, to enhance the complex interplay of postural control, mobility, proprioception, strength, coordination, agility, and endurance (see Figure 2B). These steps align with basic motor development abilities, though not all may be affected by injury. A thorough evaluation is necessary to identify which abilities have undergone reversible involution and require re-education or readaptation.

### 4.1 Postural control

Postural control provides a strong foundation through proper technique and stability. This phase focuses on correcting asymmetries, improving balance, and managing pain to support effective rehabilitation. The goal is to achieve an optimal, pain-free position through targeted exercises and interventions, establishing a solid base for the next stages of recovery.

### 4.2 Mobility

Mobility plays a crucial role in injury rehabilitation, encompassing factors such as range of motion, muscle balance, and motor control. This phase focuses on targeted exercises to enhance flexibility and correct imbalances. Techniques like tissue softening and fascia release help create a balanced musculoskeletal system, optimizing the body's ability to move efficiently.

### 4.3 Proprioception

This stage focuses on proprioception—the body's awareness of its position in space. Proprioceptive skills are developed through targeted exercises and activities that enhance body control and spatial awareness. Systematic progression and proprioceptive challenges help restore movement confidence, reduce the risk of re-injury, and improve functional capacity.

### 4.3 Strength

The third approach focuses on complex tasks, movement efficiency, and effectiveness. Strength development is a key foundation of injury recovery. This stage involves progressing from simple exercises to more challenging activities, improving both movement efficiency and effectiveness. Personalized strength training programs target specific weaknesses and imbalances, building a solid foundation for physical resilience.

### 4.4 Coordination and agility

The coordination and agility phase emphasizes neuromuscular control, reaction time, and dynamic movements such as accelerations, decelerations, landings, and jumps. Through diverse drills and exercises, individuals refine motor skills and improve their ability to adapt to changing movement demands. This stage not only aids in injury prevention but also enhances overall athletic performance.

### 4.5 Endurance

Endurance focuses on sustaining performance over time. This phase involves implementing strategies to reduce and manage fatigue, preventing performance decline and compensatory movements. The goal is to optimize recovery between efforts while building strength, allowing individuals to maintain activity for extended periods without compromising form or risking overuse injuries.

A holistic approach is maintained throughout each phase by integrating cognitive, technical, physical, metabolic, and tactical elements. Cognitive exercises enhance mental resilience, focus, and decision-making. Technical skills specific to activities or sports are continuously refined. Metabolic considerations ensure optimal energy management during rehabilitation. Tactical planning aligns the recovery process with individual goals and activity demands. This integrated approach creates a comprehensive, personalized rehabilitation program that addresses all aspects of injury recovery and readaptation.

## 5 Future challenges and research

The concept of reversible involution in sports injuries marks a revolutionary shift in the paradigms of rehabilitation and recovery. This innovative approach opens up exciting opportunities for further research, promising significant advancements in recovery and performance optimization for athletes.

We outline the following key areas for future exploration in this topic:

• Molecular and Cellular Mechanisms, a deeper understanding of the molecular and cellular processes underlying reversible involution in sports injuries is essential. Research efforts should focus on elucidating the mechanisms that regulate the regenerative potential of injured tissues. Investigating signaling pathways and genetic factors will provide valuable insights into how the body achieves natural recovery and returns to an optimal functional state. Additionally, there is a notable gap in research validating allostatic load indexes in sports-specific contexts. Further studies are needed to explore how molecular and biomarker-based allostatic load measures can be applied to assess stress and recovery in athletes, particularly in relation to injury and rehabilitation. For further guidance, we encourage readers to access the 4R's of Sports Nutrition and newly developed allostatic load indices for athletes (28);
• Personalized Rehabilitation Strategies, The reversible involution model highlights the potential for tailoring rehabilitation strategies to an athlete's unique physiological and genetic profile. Future research should prioritize the development of personalized rehabilitation protocols that account for factors such as age, sex, genetic predispositions, and injury mechanisms. Such individualized approaches could lead to more effective and cost-efficient recovery plans, minimizing downtime and optimizing an athlete's return to peak performance;
• Integration of Technology, the rise of wearable technology, bioinformatics, and artificial intelligence presents unprecedented opportunities to monitor, analyze, and enhance the rehabilitation process. Future studies should explore the integration of these technologies into real-time rehabilitation systems that adapt to the needs of athletes and professionals. A data-driven approach could improve the precision of rehabilitation strategies and increase adherence to personalized protocols;
• Psychosocial Dimensions, the psychosocial aspects of sports injuries and recovery warrant greater attention. Research should examine how reversible involution influences an athlete's psychological state, motivation, and resilience. By addressing both the physical and psychological components of recovery, holistic rehabilitation interventions can be developed to support athletes more effectively;
• Long-Term Implications, research should extend beyond the short-term outcomes of rehabilitation to investigate the long-term effects of reversible involution on an athlete's health and performance. Understanding how the body adapts to previous injuries and the implications for future injury risk is critical. This knowledge can inform injury prevention strategies, helping athletes sustain their careers and maintain peak performance over the long term.

## 6 Conclusions

The concept of reversible involution reshapes how we approach sports injury rehabilitation, emphasizing the dynamic interplay between physical recovery and psychological well-being. Viewing sports injuries as reversible involution offers a transformative framework for rehabilitation and readaptation. By understanding the allodynamic responses of athletes, the progression to allostatic overload and cacostasis, and the process of tissue involution, practitioners can design targeted strategies to optimize recovery and restore pre-injury performance levels. Addressing the physical, emotional, and psychological aspects of an athlete's life enables recovery practitioners to adopt a more integrative and holistic approach. This comprehensive perspective not only enhances physical rehabilitation but also supports psychological resilience, empowering athletes to overcome injuries and reach optimal return-to-play.
